# Utility and Limitations of TALLYHO/JngJ as a Model for Type 2 Diabetes–Induced Bone Disease

**DOI:** 10.1002/jbm4.10843

**Published:** 2023-11-17

**Authors:** Lejla Emini, Juliane Salbach‐Hirsch, Johannes Krug, Katharina Jähn‐Rickert, Björn Busse, Martina Rauner, Lorenz C. Hofbauer

**Affiliations:** ^1^ Department of Medicine III and Center for Healthy Aging Technische Universität Dresden Medical Center Dresden Germany; ^2^ Department of Osteology and Biomechanics University Medical Center Hamburg‐Eppendorf Hamburg Germany; ^3^ Mildred Scheel Cancer Career Center Hamburg University Cancer Center Hamburg, University Medical Center Hamburg‐Eppendorf Hamburg Germany; ^4^ Interdisciplinary Competence Center for Interface Research (ICCIR) University Medical Center Hamburg‐Eppendorf (UKE) Hamburg Germany

**Keywords:** ANIMAL MODELS, BONE FRAGILITY, BONE MICROARCHITECTURE, OSTEOCYTE, SWR/J, TALLYHO/JNGJ, TYPE 2 DIABETES

## Abstract

Type 2 diabetes mellitus (T2DM) increases risk of fractures due to bone microstructural and material deficits, though the mechanisms remain unclear. Preclinical models mimicking diabetic bone disease are required to further understand its pathogenesis. The TALLYHO/JngJ (TH) mouse is a polygenic model recapitulating adolescent‐onset T2DM in humans. Due to incomplete penetrance of the phenotype ~25% of male TH mice never develop hyperglycemia, providing a strain‐matched nondiabetic control. We performed a comprehensive characterization of the metabolic and skeletal phenotype of diabetic TH mice and compared them to either their nondiabetic TH controls or the recommended SWR/J controls to evaluate their suitability to study diabetic bone disease in humans. Compared to both controls, male TH mice with T2DM exhibited higher blood glucose levels, weight along with impaired glucose tolerance and insulin sensitivity. TH mice with/without T2DM displayed higher cortical bone parameters and lower trabecular bone parameters in the femurs and vertebrae compared to SWR/J. The mechanical properties remained unchanged for all three groups except for a low‐energy failure in TH mice with T2DM only compared to SWR/J. Histomorphometry analyses only revealed higher number of osteoclasts and osteocytes for SWR/J compared to both groups of TH. Bone turnover markers procollagen type 1 N‐terminal propeptide (P1NP) and tartrate‐resistant acid phosphatase (TRAP) were low for both groups of TH mice compared to SWR/J. Silver nitrate staining of the femurs revealed low number of osteocyte lacunar and dendrites in TH mice with T2DM. Three‐dimensional assessment showed reduced lacunar parameters in trabecular and cortical bone. Notably, osteocyte morphology changed in TH mice with T2DM compared to SWR/J. In summary, our study highlights the utility of the TH mouse to study T2DM, but not necessarily T2DM‐induced bone disease, as there were no differences in bone strength and bone cell parameters between diabetic and non‐diabetic TH mice. © 2023 The Authors. *JBMR Plus* published by Wiley Periodicals LLC on behalf of American Society for Bone and Mineral Research.

## Graphical abstract

Exploring the TALLYHO/JngJ mouse model in diabetic bone disease.

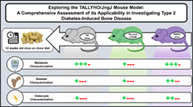

## Introduction

Type 2 diabetes mellitus (T2DM) is a rapidly growing global health issue. Due to the general population aging, the prevalence is increasing and will impose an overwhelming burden on healthcare systems. It is estimated that 463 million people are currently living with diabetes.^[^ ^1^ ^]^ T2DM accounts for 90% of diabetic cases and is characterized by hyperglycemia and insulin resistance.^[^ ^2^ ^]^ Patients with T2DM have increased risk for bone fractures despite having normal to increased bone mineral density (BMD).^[^ ^3^, ^4^, ^5^, ^6^ ^]^ Increased fall risk, obesity, duration of the diabetic disease, poor glycemic control, and other comorbidities are likely to contribute to the increased fracture risk.^[^ ^7^ ^]^ In recent years, studies have emphasized the negative impact of T2DM on bone quality and bone strength, resulting in the recognition of diabetic bone disease as a significant complication of longstanding T2DM. However, despite comprehending the underlying causes of diabetic bone disease, the mechanisms of actions are not completely understood. Thus, future research needs to better define the mechanisms underlying increased fracture risk in T2DM and optimal strategies and treating those at high risk.

The pathophysiological mechanisms underlying bone fragility in T2DM are complex and include low bone turnover,^[^ ^8^ ^]^ micro‐ and macro‐architecture alterations, and tissue material damage.^[^ ^9^, ^10^, ^11^, ^12^ ^]^ Moreover, cortical porosity is another feature of diabetic bone, in particular at the distal radius.^[^ ^13^, ^14^, ^15^, ^16^, ^17^ ^]^ Other determinants of bone fragility include inflammation, oxidative stress, WNT signaling dysregulation, and the accumulation of advanced glycation end‐products that overall compromise the bone quality aspects and increase the risk of bone fractures in patients with T2DM. Osteocytes have also been shown to be negatively impacted by T2DM.^[^ ^18^, ^19^, ^20^, ^21^, ^22^ ^]^ Previous studies have demonstrated increased lacunar density and reduced osteocyte territorial matrix in a rat T2DM model, which is the mineralized matrix volume per osteocyte lacunar.^[^ ^22^, ^23^, ^24^ ^]^ The osteocyte lacune size and the pericellular area have been reported to be reduced in diabetic pigs as well.^[^ ^25^ ^]^ Others have reported alterations of the osteocyte network in mouse model of high‐fat induced T2DM and revealed the perilacunar mineralization heterogeneity was decreased.^[^ ^26^ ^]^ The integrity of the osteocyte lacunocanalicular system (LCS) in individuals with T2DM can potentially interfere with osteocyte mechanotransduction, thereby compromising bone health and increasing risk of fractures.

Even though several important insights into diabetic bone disease have been gained by studying animal models, most animal models of T2DM do not fully recapitulate the human disease, as they often show lower BMD instead of higher BMD. Thus, there is still a need to develop and/or characterize animal models that faithfully represent T2DM bone disease in humans.

In this study, we re‐evaluated and deeply characterized the TALLYHO/JngJ (TH) mouse, which has been previously used as a model of diabetic bone disease. By the age of 10–14 weeks, hyperglycemia, insulin resistance, hyperinsulinemia, and hyperlipidemia are present.^[^ ^27^, ^28^ ^]^ TH is a polygenic model recapitulating human early‐onset T2DM and obesity. Although polygenic models offer a more accurate model of human condition unlike monogenic models, there is no proper wild‐type control for comparison. Previous studies have used the SWR/J strain as control, because it shares 86.8% of their genotype and 67.1% of their haplotype. Others have compared TH mice to C57BL/6 mice.^[^ ^29^, ^30^, ^31^ ^]^ When comparing TH to SWR/J, they show lower bone formation markers, lower trabecular volume, thicker cortices, lower bone toughness, and reduced fracture resistance, suggesting that skeletal acquisition and bone integrity is impaired in TH mice.^[^ ^32^, ^33^, ^34^ ^]^ However, due to significant differences in body size and body fat, the skeletal comparison of TH mice to SWR/J is confounded. To circumvent this issue, a previous study made use of the incomplete penetrance of the phenotype in male TH mice.^[^ ^35^ ^]^ Not all TH mice develop T2DM; those that maintain normal levels of glucose (~25%) can be used as a proper strain‐matched control. Also in that study, they examined the skeletal differences between TH mice either with or without hyperglycemia. In doing so, TH mice with diabetes had lower body weight and exhibited decrements in cortical and trabecular bone structure, along with an increase in cortical porosity, compared to nondiabetic TH mice. However, it should be noted that a very large number of animals was used to reach statistical significance.

We characterized the metabolic and skeletal phenotype of diabetic TH mice and compared them to either their nondiabetic TH controls or the recommended SWR/J controls to re‐evaluate the suitability of the TH mouse to represent diabetic bone disease in humans.

## Materials and Methods

### Animal models

Animal procedures were approved by the institutional Animal Care Committee of the Technische Universität Dresden and the Landesdirektion Sachsen (TVV 20/2017). Twelve‐week‐old male mice TALLYHO/JngJ (JAX#5314) and SWR/J (JAX#0689) were purchased from The Jackson Laboratory (Bar Harbor, ME, USA) and housed under institutional guidelines. Animals were maintained in groups of up to five animals in a light–dark cycle of 12/12 h at room temperature in filter‐top cages and had ad libitum access to drinking water and a standard chow diet (SNIFF#V1534‐300). Within our study we used a total of 20 diabetic TH mice, 20 of the recommended control SWR/J and 20 nondiabetic TH mice. The weight and blood glucose were measured with a scale and a glucometer (ACCU CHECK aviva III; Roche Diabetes Care, Mannheim, Germany) once a week for the time course of 12 and 20 weeks to establish mice developed T2DM. Those mice with nonfasting blood glucose levels >12 mmol/L were considered diabetic whereas those with normal nonfasting glucose levels <12 mmol/L were defined nondiabetic.^[^ ^36^ ^]^ After 12 and 20 weeks of age, mice were euthanized using CO_2_. Blood was collected via heart puncture and serum was obtained by centrifugation. For subsequent bone analyses, fourth lumbar vertebrae (L_4_) and femurs were collected postmortem, fixed in 4%buffered paraformaldehyde for 48 h and stored in 50% ethanol. Further tissues (femur, tibia, and bone marrow) were collected and cryoconserved for subsequent analysis.

### Intraperitoneal glucose tolerance test

Twelve‐week‐old TH and SWR/J were fasted overnight and D‐glucose (Sigma‐Aldrich, St. Louis, MO, USA) dissolved in phosphate‐buffered saline (PBS) was administered intraperitoneally at 2 g/kg body weight to assess their glucose tolerance. Fasted blood glucose levels were measured in tail vein using a glucometer immediately before and 15, 30, 60, 90, and 120 min after glucose administration. During intraperitoneal glucose tolerance test (ipGTT) mice had ad libitum access to their drinking water.

### Intraperitoneal insulin tolerance test

Twelve‐week‐old TH and SWR/J were fasted 6 h prior to the intraperitoneal injection of insulin to assess insulin tolerance. Mice were administered 0.4 IE/kg body weight insulin (Huminsulin®, 100 IE/mL; Lilly Deutschland GmbH) and fasted blood glucose levels were measured immediately before and subsequently at 15, 30, 60, 90, and 120 min after insulin administration. During intraperitoneal insulin tolerance test (ipITT) all mice had ad libitum access to their drinking water.

### Clinical chemistry

Blood was collected and centrifuged for 20 min at 10,000*g* at 4°C to collect serum. The concentration of bone turnover markers procollagen type 1 amino‐terminal propeptide (P1NP), C‐terminal telopeptide of type 1 collagen (CTX), and tartrate‐resistant acid phosphatase (TRAP) were assessed using enzyme‐linked immunosorbent assays (ELISAs) according to the manufacturer's protocol (IDS, Frankfurt/Main, Germany). Insulin levels were determined by ELISA according to the manufacturer's protocol (Thermo Fisher Scientific, Bonn, Germany). Levels of triglyceride, urea and cholesterol were determined by ELISA at the Institute für Klinische Chemie und Laboratoriumsmedizin (IKL, Dresden, Germany).

### Microarchitecture analysis by micro–computed tomography

To determine bone mass and bone microarchitecture, the distal femur and fourth lumbar vertebra (L_4_) were analyzed using micro–computed tomography (μCT) (vivaCT40; Scanco Medical, Brüttisellen, Switzerland) with an X‐ray energy of 70 kVp and isotropic voxel size of 10.5 μm (114 mA, 200 msec integration time). Trabecular (Tb.) and cortical (Ct.) bone parameters including bone volume/total volume (BV/TV), trabecular number (Tb.N), trabecular separation (Tb.Sp), and trabecular thickness (Tb.Th) were assessed based on calculations including 100 scan slices following standard protocols from Scanco Medical. Trabecular parameters of femur were assessed in the metaphyseal region starting 20 slices below the growth plate, whereas cortical bone analyses were performed within the diaphyseal region midway between femoral head and distal condyles. Trabecular bone of L_4_ was evaluated at the center contouring 50 slices above and 50 slices below the middle of the vertebral body.

The femur length was measured postmortem using a caliper.

### High‐resolution μCT of osteocyte lacunar

To image osteocyte lacunar in bone, the femur and vertebral (L_4_) body were imaged with a μCT50 (Scanco Medical AG, Brüttisellen, Switzerland), operated with 0.5 mm aluminum filter, 57 μA current, 4 W power, 70 kVp energy, 3500 ms integration time, level 2 data averaging, and with a total of 1500 projections per 180 degrees. Images were reconstructed at a nominal isotropic voxel size resolution of 1.0 μm with an anti‐ring level 2 to minimize center ring artifacts using the manufacturer's scanner software. Each image consisted of a cylindrical volume with a radius of 4.1 mm (containing the full sample) and the height of one scan stack (1103 slices = 1.10 mm). The protocol for each sample consisted of two scans: (i) prescan to warm the sample in the scanner gantry in an effort to reduce motion artifacts caused by thermal effects; and (ii) a scout view was made to select region of interest (ROI). The reference line was set with a fixed distance of 2.6 mm starting below the growth plate until the midshaft to assure the same region was selected for all femurs.

### Assessment of lacunar and porosity indices using μCT

Five hundred slices (0.5 mm) from the distal part of the scan and 500 slices of the proximal part of the scan toward the midshaft were selected to investigate the trabecular and cortical lacunar morphology in the femur, respectively. Cortical and trabecular regions were segmented automatically using a series of morphological and logical operations after applying a pre‐threshold,^[^ ^37^ ^]^ which were used to mask the original gray values images. For lacunar analysis in the vertebrae, masks were created by manually contouring the trabecular bone of 300 consecutive slices (0.3 mm) in the vertebral body. Subsequently, an individual threshold based on the method proposed by Goff et al.^[^ ^38^ ^]^ was applied to all masked compartments to account for differences in mineralization. In the binarized images, closed holes with a volume of 100–2000 μm^3^ were assumed to be lacunar, wheras open pores with a volume >2000 μm^3^ were counted as vascular canals. Lacunar size and number were quantified by measuring lacunar number density (N.Lc/BV), volume density (Lc.V/BV), and individual mean lacunar volume (Lc.V/N.Lc). Lacunar shape was described by lacunar sphericity (Lc.Sph), stretch (Lc.St), oblateness (Lc.Ob), and orientation of the longest axis in regard to the longitudinal axis of the bone (Lc.Θ), based on ellipsoid fit to the lacunar.^[^ ^39^ ^]^ For the cortical vascular canal system, canal porosity (Ca.V/Ca.TV), number density (N.Ca/Ct.TV), diameter (Ca.Dm), and separation (Ca.Sp) were evaluated. Visual inspection of all samples revealed successful segmentation of cortical and trabecular compartments, but also lacunar and vascular canal parameters Image segmentation, analysis and visualization were performed in XamFlow (Lucid Concept AG, Zurich, Switzerland). For automatic thresholding and lacunar analysis, custom Python 3.10 scripts (Python Software Foundation, Beaverton, USA) were implemented into the software.

### Biomechanical testing

Femurs were used for a three‐point bending test to assess cortical bone strength (Zwick Roell, Ulm, Germany). Femurs were rehydrated in PBS overnight and placed onto two supports with an intermediate distance of 6 mm. Mechanical force was applied vertically on to the middle of the femoral midshaft. After reaching a preload of 1 N, the measurement started and continued with a load rate of 0.05 mm/s until failure.

The maximum strength of the fifth lumbar (L_5_) vertebrae was evaluated via a compression test. The vertebrae were cleaned from muscles and ligaments. These specimens were placed on a flat surface in a material testing machine (Zwick Roell, Ulm, Germany) and a compression force by another flat plunger was applied with a preload of 0.5 N and held with a load rate of 0.05 mm/s until failure. The maximal load (F_max_) and energy to failure were quantified as an indicator of bone strength and stiffness, respectively using testXpert II‐ V3.7 software (Zwick Roell, Ulm, Germany).

### Bone histomorphometry

Two intraperitoneal injections of calcein (20 mg/kg) were injected 5 days and 3 days prior to euthanasia. Femur and the third lumbar (L_3_) were fixated in 4% PBS‐buffered paraformaldehyde and dehydrated in ascending ethanol series. Subsequently, bones were embedded in methylmethacrylate (Technovit 9100; Heraus Kulzer, Hanau, Germany) and cut into 7‐μm sections to assess fluorescence labels. Sections were analyzed using fluorescence microscopy to determine mineral apposition rate (MAR), mineral surface per bone surface (MS/BS), and the bone formation rate/bone surface (BFR/BS) using the two fluorescent labels. Histomorphometry analysis was performed with the Osteomeasure software (Osteometrics, Decatur, GA, USA) according to the international standards.^[^ ^40^ ^]^

Femurs and the fourth lumbar vertebrae (L_4_) were isolated, fixated with 4% paraformaldehyde for 48 h and decalcified in Osteosoft (Merck, Germany) over a period of 7 days and dehydrated using ascending ethanol series. Decalcified bones were embedded in paraffin. Sections of 2 μm were prepared and stained with tartrate‐resistant acid phosphatase (TRAP). TRAP staining was performed to quantify the number of osteoclast per bone perimeter (N.Oc/B.Pm), the number of osteoblast (N.Ob/N.Pm), as well as the number of osteocytes per bone perimeter (N.Ocy/B.Pm) in an area of 0.90 mm^2^ and 0.48 mm^2^ in the center of vertebrae and femoral metaphysis respectively, using the Microscope Axio Imager M1 (Carl Zeiss Jena, Jena, Germany) and Osteomeasure software (Osteometrics). Furthermore, the number of adipocytes per bone perimeter (N.Adi/B.Pm) was also assessed. We evaluated one slide per animal.^[^ ^41^ ^]^ Representative photos were taken using CellSens Entry Software Version 1.5 (OLYMPUS Cooperation, Shinjuku, Japan).

### Osteocyte canaliculi evaluation

For osteocyte canaliculi evaluation, 4‐μm sections of femoral and vertebral bone embedded in methylmethacrylate (MMA) were stained using Ploton's silver nitrate precipitation.^[^ ^42^ ^]^ With this, the osteocyte canaliculi becomes visible and allows access to two‐dimensional (2D) canalicular number (Ot.Ca/Lc) and the osteocyte lacunar area (Ot.Ca/Lc.Ar, 1/μm^2^). Compartment‐specific analyses were performed in the mid‐cortical bone region for cortical analysis and in the trabecular compartment 1 mm below the growth plate. Per sample and region 15 osteocytes lacunar were evaluated.

### Acid etching and scanning electron microscopy

Acid etching was performed on femur and vertebrae specimens. The embedded specimens were polished using an automatic grinding system (Exakt, Germany) to obtain flat coplanar surfaces. Polished samples were immersed in 9% phosphoric acid for 20 s, rinsed in deionized water and exposed to 5% sodium hypochlorite. Specimens were dried and sputter coated with gold alloy and evaluated by scanning electron microscopy (LEO 435 VP; LEO Electron Microscopy Ltd., Cambridge, UK).

### Statistical analysis

Results are presented as mean ± standard deviation (SD). Differences between all three groups were evaluated by one‐way analysis of variance (ANOVA) followed by Bonferroni's multiple comparison post‐hoc test using GraphPad Prism 9.0 0 (GraphPad, La Jolla, CA, USA). For both ipITT and ipGTT, the area under the curve (AUC) was generated to compare the levels of glucose among the experimental groups.

Data are presented as the mean ± SD, values of *p* < 0.05, *p* < 0.01, or *p* < 0.001 were considered statistically significant, unless stated otherwise. Significant outliers were excluded based on Grubbs' test provided in GraphPad by Dotmatics (https://www.graphpad.com/quickcalcs/grubbs1/).

## Results

### Metabolic characterization of TH mice

**Fig. 1:**
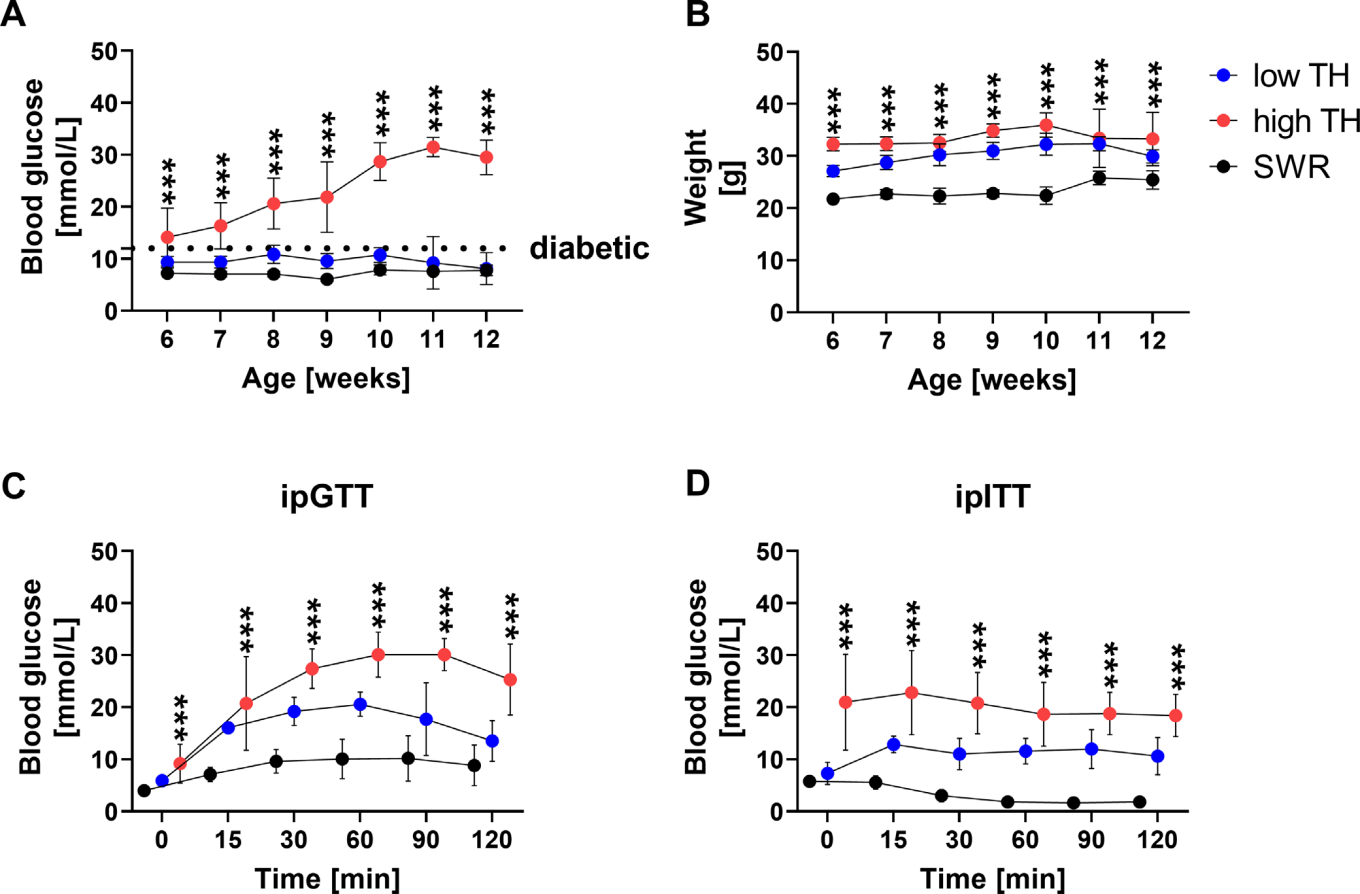
Metabolic parameters show differences between diabetic TH (high TH) and nondiabetic (low TH). (*A*) Nonfasting blood glucose levels and (*B*) weight were measured for TH and SWR/J male mice once a week starting from 6 weeks of age until 12 weeks of age. (*C*) ipGTT, the glucose tolerance was assessed by intraperitoneal glucose tolerance test in 12‐week‐old mice. D‐glucose dissolved in PBS was administered 2 g/kg body weight. Fasted blood glucose levels were measured immediately before and 15, 30, 60, 90, and 120 min after glucose administration. (*D*) Insulin tolerance was investigated by intraperitoneal insulin tolerance test (ipITT) in 12‐week‐old mice. 0.4 IU/kg body weight insulin was administered and fasted blood‐glucose levels were measured immediately before and subsequently at 15, 30, 60, 90, and 120 min after. Data are shown as mean ± SD. *n* = 5. Statistical analysis was performed by one‐way ANOVA followed by Bonferroni correction. Statistical significance is denoted in the graphs. *p < 0.05, **p < 0.001, ***p < 0.001.

Diabetes is defined by nonfasting glucose levels 12 mmol/L or greater in mice.^[^ ^43^, ^44^ ^]^ Comparison of diabetic TH (high‐TH) versus its recommended control SWR/J and nondiabetic TH (low‐TH) used as a strain‐matched control revealed blood glucose levels were significantly higher (+69%, *p* < 0.001 vs. SWR/J and +58% vs. low‐TH, *p* < 0.001) in TH mice exhibiting hyperglycemia compared to both controls, respectively. The degree of hyperglycemia progressively elevated from onset (~6 weeks) until the end of the 12‐week period (Fig. 1A). High‐TH mice displayed a higher body weight as early as 6 weeks of age compared to SWR/J (+40%, *p* < 0.001) and low‐TH (+10%, *p* = 0.004) and was maintained for the 12‐week study period (Fig. 1B). Additionally, levels of triglycerides were significantly higher in high‐TH mice compared to both control groups (+68%, *p* < 0.001 vs. SWR/J and +27%, *p* < 0.001 low‐TH) (Fig. S1A), respectively. Cholesterol and urea were significantly reduced in SWR/J compared to low‐TH mice (*p* < 0.002). No change was observed for high‐TH and low‐TH mice (Fig. S1B,C). Circulating insulin levels revealed to be significantly higher in diabetic TH compared to both controls, respectively (+47% *p* = 0.001 vs. SWR/J and +38%, *p* = 0.08 vs. low TH, Fig. S1D).

Intraperitoneal blood glucose tolerance testing indicated impaired glucose tolerance in high‐TH mice as shown by the reduced blood glucose clearance over time (Fig. 1C). Moreover, insulin tolerance testing revealed high‐TH mice displayed a whole‐body insulin resistance indicated by a significantly higher blood glucose level at baseline and maintained significantly high throughout the time course of the study compared to its respective controls (+85%, *p* < 0.001 vs. SWR/J and +46%, *p* < 0.001 vs. low‐TH) (Fig. 1D). Furthermore, low‐TH and SWR/J showed low blood glucose levels at baseline. This was more profound for SWR/J with significantly lower blood glucose at baseline compared to low‐TH (−70%, vs. low‐TH, *p* < 0.001) and maintained low throughout the time course of the study.

### Diabetic and nondiabetic TH mice have a similar trabecular and cortical bone microstructure

**Fig. 2:**
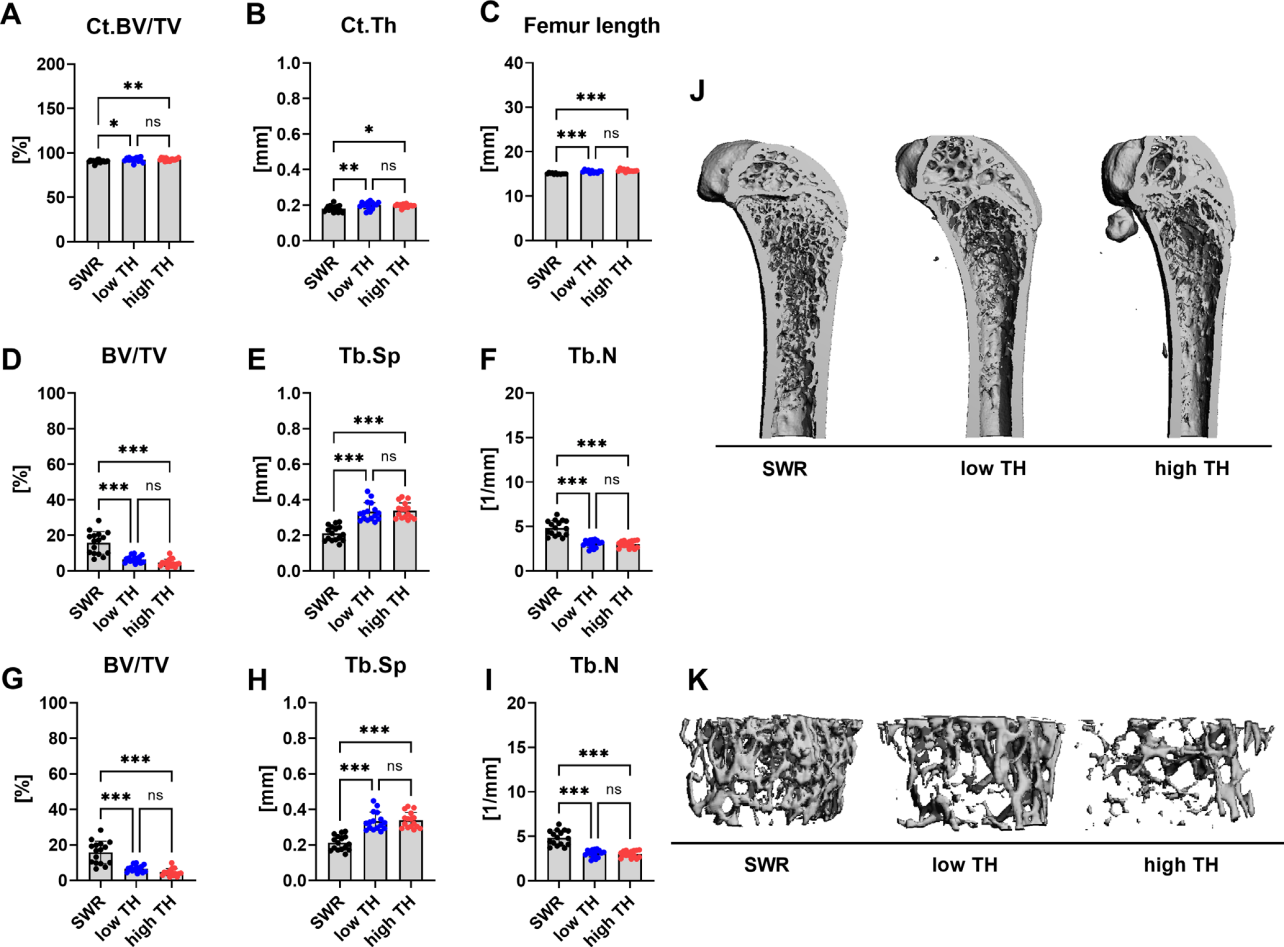
Differences in trabecular bone architecture and bone mineral density. Bones from 12‐week‐old male TALLYHO/JngJ either with hyperglycemia and without hyperglycemia and SWR/J were examined by μCT. (*A*) cortical bone volume/total volume (Ct.BV/TV) and (*B*) cortical thickness at the femoral midshaft was determined. (*C*) Femur length was measured with a caliper (*D*) bone volume per total volume (BV/TV), (*E*) trabecular separation (Tb.Sp), and (*F*) trabecular number (Tb.N) were determined in the distal femur. (*G*) bone volume per total volume (BV/TV), (*H*) trabecular spacing (Tb.Sp), and (*I*) trabecular number (Tb.N) were evaluated at L_4_ vertebrae from all groups. (*J*) Representative 3D reconstructions of the whole femur. (*K*) Representative 3D reconstructions of the trabecular compartment of L_4_ vertebrae from SWR/J and TALLYHO/JngJ with and without hyperglycemia. Data are shown as mean ± SD. *n* = 16. Statistical analysis was performed by one‐way ANOVA and Bonferroni correction. Statistical significance is denoted in the graphs. *p < 0.05, **p < 0.001, ***p < 0.001.

μCT measurement of the femur displayed high‐TH mice had elevated cortical bone volume/total volume (+2.3%, *p* = 0.005) (Fig. 2A), higher cortical bone mineral density (+7.12%, *p* < 0.001) (Fig. S2A) and cortical thickness (+9.49%, *p* < 0.001) (Fig. 2B) compared to SWR/J at the age of 12 weeks (+2.3%, *p* = 0.005) as described before.^[^ ^33^ ^]^ No changes in cortical bone were observed between TH mice with or without hyperglycemia. The femur length was shorter for the SWR/J mice compared to both subgroups of TH mice (−3.08%, *p* < 0.001 vs. low‐TH, −4.4%, *p* < 0.001 vs. high‐TH) (Fig. 2C).

At the trabecular site of the femur, high‐TH showed a lowered trabecular bone volume/total volume (BV/TV) (−70%, *p* < 0.001) (Fig. 2D) along with an increase in trabecular separation (Tb.Sp) (+36%, *p* < 0.001) (Fig. 2E) compared to SWR/J. Trabecular number (Tb.N) followed the same tendency as the BV/TV being significantly lower for high‐TH (−37%, *p* < 0.001 vs. SWR/J) (Fig. 2F). No differences were observed between the two strain‐related groups. Trabecular thickness (Tb.Th) was not different between any of the groups in both femur and spine (Fig. S2B). Similar to the femur, we found lower trabecular BV/TV (−70%, *p* < 0.001) (Fig. 2G), higher trabecular separation (+37%, *p* < 0.001) and low trabecular number (−37%, *p* = 0.001) (Fig. 2H,I) in the vertebral body observed for the high‐TH mice compared to SWR/J. Additionally, we analyzed the femurs and vertebrae from all three groups at 20 weeks old to uncover possible long‐term effects of hyperglycemia. The trabecular and cortical bone parameters for both femurs and vertebrae followed the same tendency as the group at 12‐weeks old (Fig. S3A–H). Differences between TH with and without hyperglycemia were not observed, suggesting that the different genetic background rather than the hyperglycemia state influences bone microarchitecture.

### High‐TH reveal lower bone toughness compared to SWR/J

Results obtained from μCT revealed significant differences in trabecular and cortical parameters between TH with diabetes and SWR/J mice. Thus, we next tested bone strength using a three‐point bending test for femurs and a compression test of L_4_ vertebrae.

**Fig. 3:**
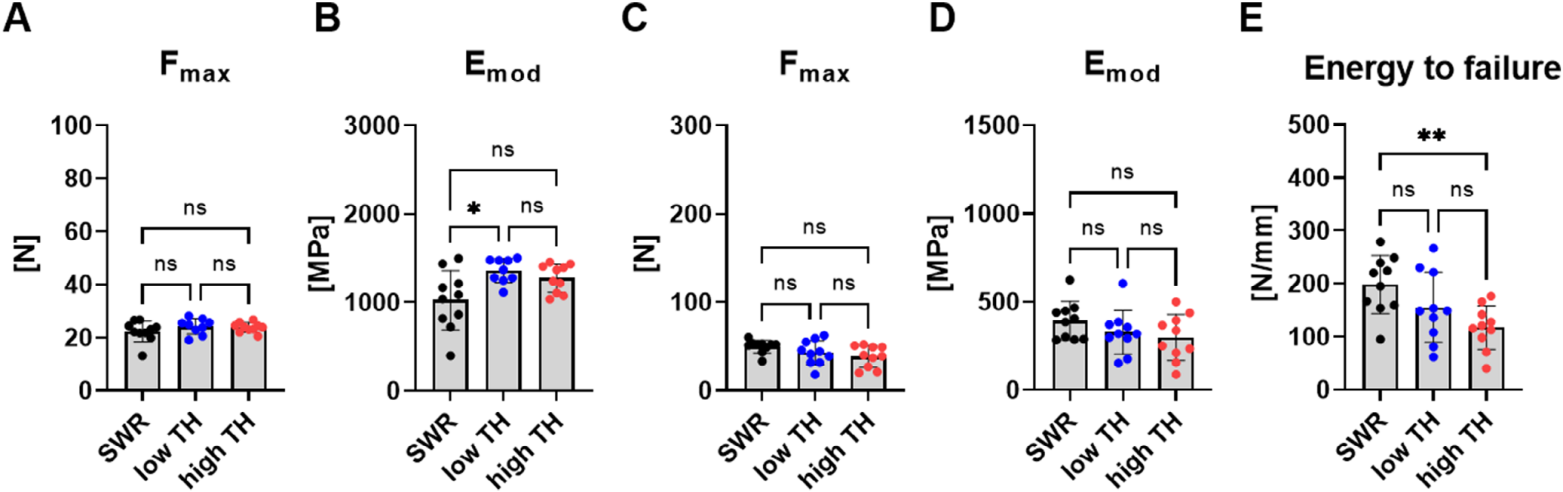
Bone mechanical properties. To assess bone strength (*A*) maximal force (F_max_) and (*B*) elastic modulus (E_mod_) was determined by three‐point bending test of femur for 12‐week‐old male TALLYHO/JngJ with and without hyperglycemia and SWR/J. (*C*) F_max_, (*D*) E_mod_, and (*E*) energy to failure was determined for the vertebrae for all three groups. Data are shown as mean ± SD. *n* = 10. Statistical analysis was performed by one‐way ANOVA and Bonferroni correction. Statistical significance is denoted in the graphs. *p < 0.05, **p < 0.001, ***p < 0.001.

Three‐point bending of femurs indicated no significant changes for Fmax or elastic modulus/stiffness in any of the three groups (Fig. 3A,B). Fmax was also not different between the groups at the L_4_ vertebra. However, high‐TH mice showed the lowest mean value of energy to failure, a parameter defining material toughness compared to SWR/J. High‐TH presented with a significantly lower energy to failure and therefore resistance to fracture (−40%, *p* = 0.008) (Fig. 3E).

### Histomorphometric analysis revealed distinct differences in trabecular bone compartments

**Fig. 4:**
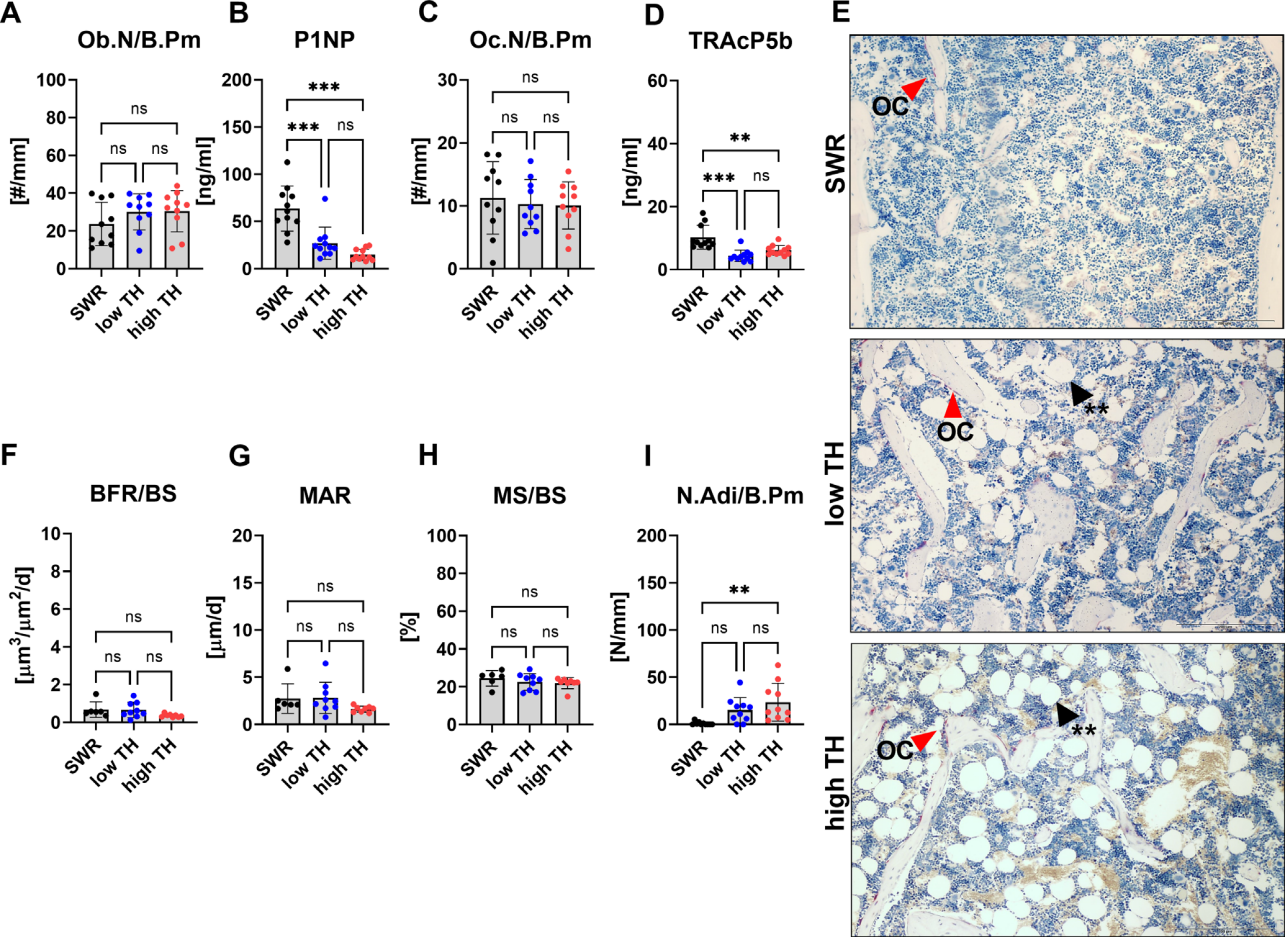
Histological and histomorphometric analysis of the femur. Bones and serum samples from 12‐week‐old TALLYHO/JngJ that either are hyperglycemic or normoglycemic and the recommended control SWR/J were used for histological analysis and determination of bone turnover markers. (*A*) Osteoblast number per bone perimeter (Ob.N/B.Pm) was determined in TRAP‐stained sections of the femur. Serum concentration of (*B*) bone formation marker type 1 procollagen amino‐terminal‐propeptide (P1NP) was measured by ELISA. (*C*) osteoclast number per bone perimeter (Oc.N/B/Pm) were determined in TRAP‐stained sections in the femur and (*D*) tartrate resistant acid phosphatase (TRAcP5b) was measured by ELISA. (*E*) Representative images of TRAP‐stained femur. Red arrows indicate osteoclast. Black arrows indicate adipocytes. (*F*) bone formation rate per bone surface (BFR/BS), (*G*) mineral apposition rate (MAR), (*H*) mineralized surface per bone surface (MS/BS), and (*I*) number of adipocytes per bone perimeter (N.Adi/B.Pm) were assessed using histomorphometry and histological analysis of the femur. Data are shown as mean ± SD. *n* = 11. Statistical analysis was performed by one‐way ANOVA and followed by Bonferroni correction. Statistical significance is denoted in the graphs. *p < 0.05, **p < 0.001, ***p < 0.001.

To assess bone remodeling, bone histomorphometric analyses of femoral and vertebral bone slices were performed. At the femur, no change was observed between all three groups in terms of osteoblast number (Fig. 4A), despite the significantly higher P1NP serum concentrations in SWR/J compared to both TH groups (+57%, *p* < 0.001 vs. low‐TH and +76%, *p* < 0.001 vs. high‐TH) (Fig. 4B). With regard to bone resorption, osteoclast number was not affected by T2DM and no differences were seen between SWR/J versus high‐TH and versus low‐TH (Fig. 4C) though an increase in TRAcP5b serum concentration was observed in the SWR/J (+58%, *p* < 0.001 vs. low‐TH and +40%, *p* = 0.002 vs. high‐TH) (Fig. 4D). All three groups at 20 weeks old revealed same tendency for the bone turnover markers. TRAcP5b and P1NP was elevated in SWR/J compared to both subgroups of TH (Fig. S4A,B). This also suggests that the bone turnover markers could reflect the lower trabecular bone mass observed for both subgroups of TH mice.

**Fig. 5:**
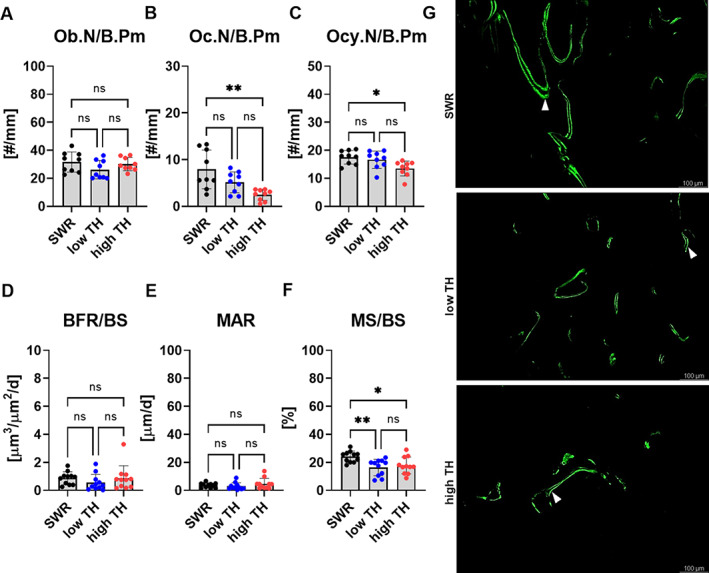
Histological and histomorphometric analysis of the vertebrae. At the age of 12 weeks, (*A*) osteoblast number per bone perimeter (Ob.N/B.Pm.), (*B*) osteoclast number per bone perimeter (Oc.N/B.Pm), (*C*) osteocyte number per bone perimeter (Ocy.N/B.Pm) from all three groups were assessed using histological analysis of vertebral slides. (*D*) Bone formation rate per bone surface (BFR/BS), (*E*) mineral apposition rate (MAR), and (*F*) mineralized surface per bone surface (MS/BS) were determined using histomorphometric analysis of the spine from all three groups. (*G*) Representative fluorescence images of calcein double labels that are indicated by white arrows. Data are shown as mean ± SD. *n* = 11. Statistical analysis was performed by one‐way ANOVA and Bonferroni correction. Statistical significance is denoted in the graphs. *p < 0.05, **p < 0.001, ***p < 0.001.

T2DM had no significant effect on the bone formation rate, mineral apposition rate, and mineralized surface (Fig. 4F–H). However, the bone marrow adipocyte number was higher in the high‐TH group in the femur (+95%, *p* = 0.004) compared to SWR/J (Fig. 4I,E). Histomorphometry of the vertebral bone revealed similar differences. The osteoblast number did not differ between any of the groups (Fig. 5A). However, elevated number of osteoclasts were seen in SWR/J compared to high‐TH in the vertebrae (+69% *p* = 0.001) (Fig. 5B). More interestingly, a low number of osteocytes was revealed for high‐TH compared to SWR/J (−22%, *p* = 0.014) (Fig. 5C). Further, the bone formation rate and mineral apposition showed no change between high‐TH compared to its respective controls (Fig. 5D,F,G). SWR/J had more mineralized surface in the spine compared to both subgroups of TH mice in the vertebrae (+31.8%, *p* = 0.007 vs. low‐TH and +25.9%, *p* = 0.031 vs. high‐TH) (Fig. 5F).

### Lower lacunar connectivity in femoral trabecular bone of high‐TH mice and more stretched lacunar morphology in TH mice

Osteocytes create and form the sophisticated canalicular network in the bone to provide for the exchange for oxygen and nutrients. Changes and disparities within this network may lead to alterations in bone remodeling and reduced bone quality. Thus, we investigated if the canaliculi network is affected in TH mice. To that end, silver nitrate staining was performed on the femur and spine.

**Fig. 6:**
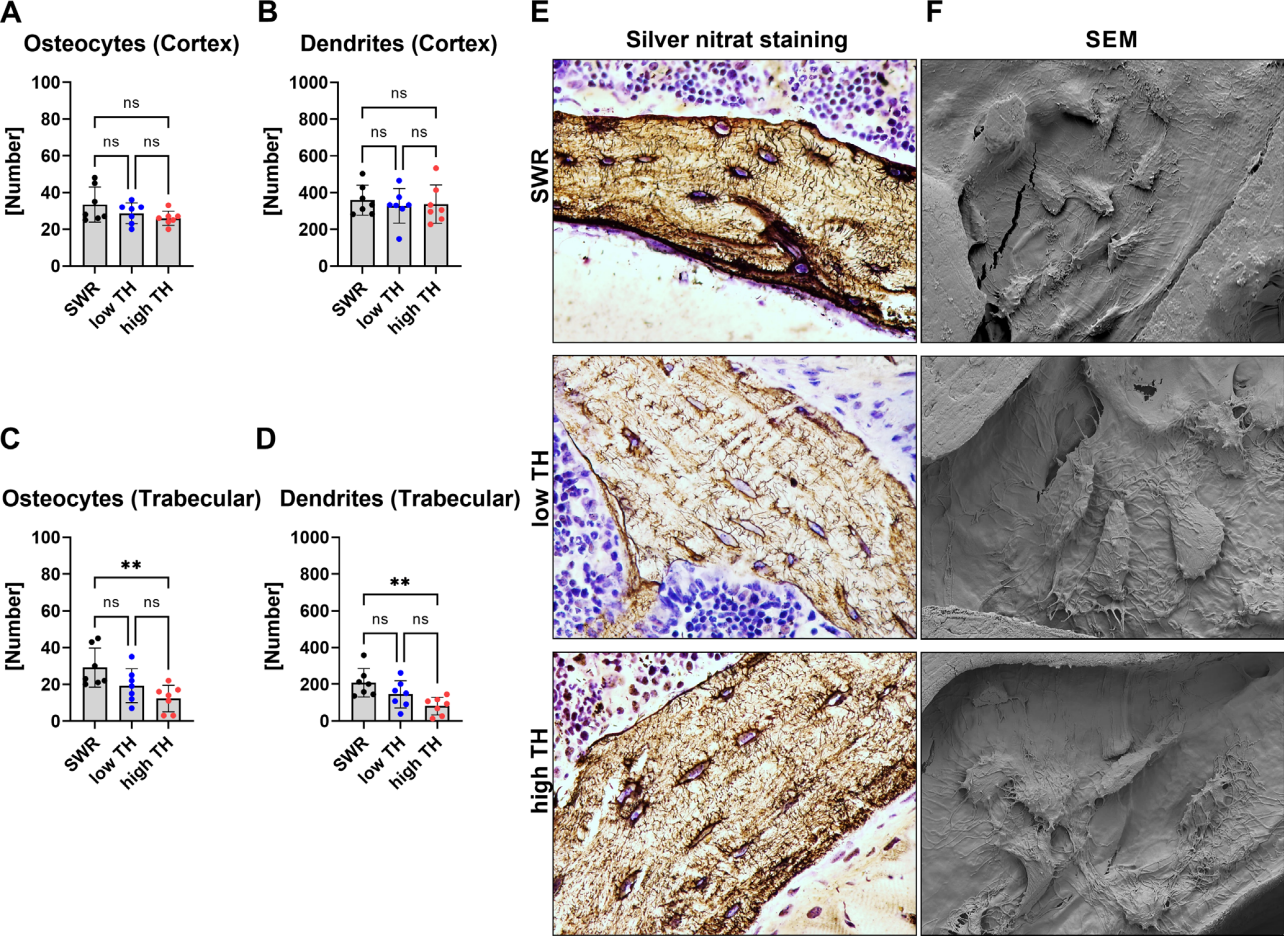
The osteocyte canaliculi network is impaired in diabetic TH. At the age of 12 weeks, (*A*) number of osteocytes and (*B*) number of dendrites in the cortex of the femur including (*C*) number of osteocytes and (*D*) number of dendrites in the trabecular bone of the femur were assessed by silver nitrate staining. (*E*) Representative images of silver nitrate stained femoral slides. (*F*) Representative images by scanning electron microscope (SEM). Data are shown as mean ± SD. *n* = 7. Statistical analysis was performed by one‐way ANOVA and Bonferroni correction. Statistical significance is denoted in the graphs. *p < 0.05, **p < 0.001, ***p < 0.001.

Of note, the number of osteocytes and the number of dendrites in the cortex revealed no differences between all three groups in the femur (Fig. 6A,B). This was also shown for the spine (Fig. S5A,B). In the trabecular bone of the femur, an augmented number of osteocytes was seen in SWR/J compared to high‐TH group (+57%, *p* = 0.008) (Fig. 6C). Moreover, SWR/J had more dendrites compared to high‐TH (+60%, *p* = 0.008) (Fig. 6D). Further, the osteocyte number and dendrite number did not change between all groups in the spine in the trabecular bone (Fig. S5C,D).

**Fig. 7:**
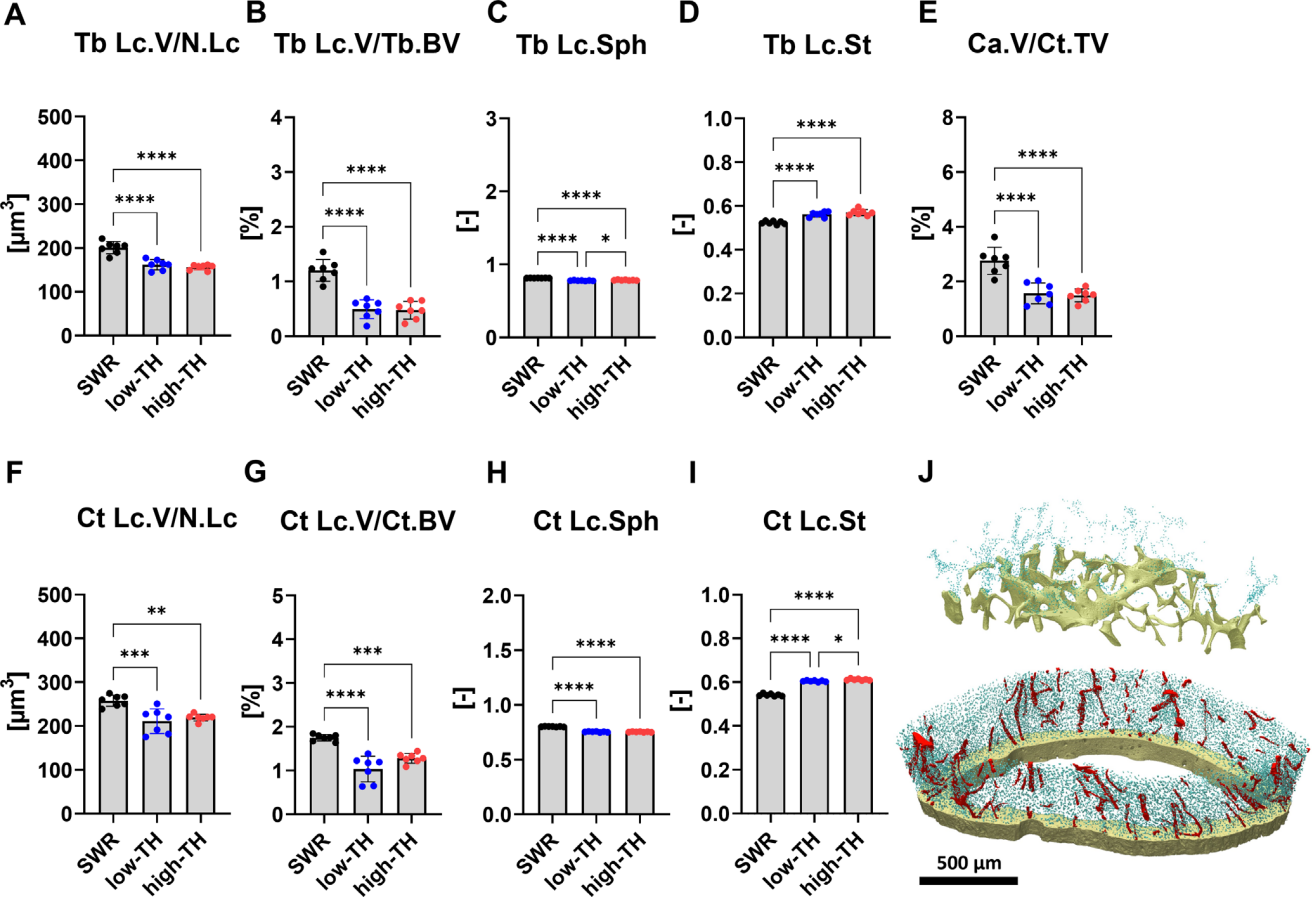
Characterization of osteocyte lacunar network. High‐resolution μCT was performed on femurs from 12‐week‐old TALLYHO/JngJ either with T2DM or without and the recommended control SWR/J. (*A*) Trabecular lacunar volume over number of lacunar (Tb.Lc.V/N.Lc) and (*B*) Trabecular lacunar volume over bone volume (Tb.Lc.V/Tb.BV). Additionally the morphology of the osteocytes was investigated. (*C*) Trabecular lacunar sphericity (Tb.Lc.Sph) and (*D*) trabecular lacunar stretch (Tb.Lc.St). (*E*) Canal volume density over canal cortical total volume (Ca.V/Ct.TV), (*F*) cortical lacunar volume over number of lacunar (Ct.Lc.V/N.Lc), (*G*) cortical lacunar volume over cortical bone volume (Ct.Lc.V/Ct.BV), (*H*) cortical lacunar sphericity (Ct.Lc.Sph), and (*I*) cortical lacunar stretch (Ct.Lc.St). (*J*) Representative 3D reconstructions of trabecular bone and cortical bone. Blue represents osteocytes and red represents pores. Data are shown as mean ± SD. *n* = 7. Statistical analysis was performed by one‐way ANOVA and Tukey test. Statistical significance is denoted in the graphs. *p < 0.0332, **p < 0.0021, ***p < 0.0002, ****p < 0.0001.

To further characterize osteocyte morphology, a comprehensive three‐dimensional (3D) evaluation of osteocyte lacunar network was conducted. In the trabecular compartment of the femur the volume of lacunar over total lacunar number and over bone volume were unaffected and no differences were seen for high‐TH and low‐TH, although the mean volume of individual lacunar (−19% *p* < 0.0001 vs. low‐TH and −24% *p* = <0.0001 vs. high‐TH) revealed to be high for SWR/J (Fig. 7A). Lacuna volume density followed the same tendency with a two‐fold increase in SWR/J (Fig. 7B).

Differences in osteocyte morphology were prominent in SWR/J compared to both subgroups of TH mice. Sphericity unveiled to be very low for high‐TH and low‐TH compared to SWR/J (−5.5% *p* < 0.0001 vs. low‐TH and −3.7% *p* < 0.0001 vs. high‐TH) (Fig. 7C), which was further confirmed by an increase in high‐TH and low‐TH compared to the SWR/J (+6.7% *p* = <0.0001 vs. low‐TH and +8% *p* < 0.0001 vs. high‐TH) (Fig. 7D), indicating a more elongated shape. In the cortical bone compartment of the femur, canal volume density was significantly lower in both subgroups of TH mice (−76%, *p* < 0.0001 in low‐TH and −84%, *p* < 0.0001 in high‐TH) compared to SWR//J (Fig. 7E).

Further, the volume of lacunar over total lacunar number in the cortical bone exhibited a similar trend as observed for the trabecular compartment. No change was seen between low‐TH and high‐TH in regard to mean individual lacunar volume only when compared to SWR/J. It was very prominent that SWR/J showed elevated lacunar volume (−22%, *p* = 0.0005 vs. low‐TH and −17%, *p* = 0.0029 vs. high‐TH) (Fig. 7F). Lacunar volume density in the cortex was significantly lower in both subgroups of TH (−68%, *p* = <0.0001 vs. low‐TH and −36%, *p* = 0.0005 vs. high‐TH) (Fig. 7G).

In addition, the lacunar morphology in the cortical compartment showed that the osteocyte lacunar are less spherical compared to SWR/J (−5.5%, *p* < 0.0001 vs. low‐TH and −3.7%, *p* < 0.0001 vs. high‐TH) (Fig. 7H) but are more stretched in both subgroups of TH mice (+6.7%, *p* < 0.0001 vs. low‐TH and 8%, *p* < 0.0001 vs. high‐TH) compared to SWR/J (Fig. 7I). No change was seen for all three groups in terms of individual lacunar volume (Fig. S6A), although lacunar volume density was higher for SWR/J only in comparison to TH mice with hyperglycemia (+23%, *p* = 0.0152) (Fig. S6B).

The osteocyte morphology in the vertebral body showed similar effects as we observed for the femur with osteocyte lacunar being less spherical in both subgroups of TH mice (−3%, *p* = 0.0002 vs. low‐TH and −2.9%, *p* = 0.0017 vs. high‐TH). Although they were more stretched in both subgroups of TH mice compared to SWR/J (+5.5%, *p* = 0.0095 vs. low‐TH and 5.3%, *p* = 0.0133 vs. high‐TH) (Fig. S6C,D).

## Discussion

The use of preclinical rodent models that recapitulate human diabetic bone disease is crucial to gain deeper mechanistic insights that could be gained from human samples. Although rodents are commonly used to study T2DM, it is important to acknowledge that they do not perfectly replicate the complexities of the disease as observed in humans. One significant difference lies in the genetic basis of T2DM. In humans, T2DM is predominantly polygenic with a slow onset during adulthood, which is also greatly influenced by diet and physical exercise. In contrast, T2DM in rodents often involve the manipulation of specific genes (leptin or leptin receptor) or extreme variations in diet to induce the disease rapidly. This usually comes at the cost of inducing T2DM at a young age, which poorly reflects the human situation. Nonetheless, preclinical models offer valuable insights into disease mechanisms, although results must be interpreted carefully for human translation.

In this study, we deeply characterized the bone phenotype of the polygenetic TH model on a standard diet and re‐evaluated its utility as a model to study diabetic bone disease. Previously, studies have reported skeletal and metabolic differences in TH mice with T2DM compared to the recommended control group SWR/J without T2DM. However, TH and SWR/J mice are different mouse strains that only share a certain proportion of genetics (only about 86.8%) and are rather different. This suggests a complete lack of a similar genetic background. This is reflected by the different size, weight, and femur length of the two mouse strains. Thus, to avoid comparing apples with pears, we included a strain‐matched normoglycemic TH control, taking advantage of the incomplete penetrance of T2DM in TH male mice. This allowed us to perform a deep characterization of the metabolic milieu and the skeletal phenotype of diabetic TH mice compared to both, their nondiabetic TH controls or the SWR/J, to evaluate their suitability to represent diabetic bone disease in humans.

Hyperglycemia in high‐TH mice started at 6 weeks of age and progressively increased until 20 weeks of age. Thus, the onset of hyperglycemia in these mice occurs much earlier than in humans with T2DM, potentially even affecting processes of bone development. Moreover, TH mice are heavier than SWR/J mice, with minor differences between diabetic and nondiabetic TH mice. The obesity that TH mice display is not as severe as seen for other polygenic murine strains that have been reported such as NZO and TSOD mouse model^[^ ^45^, ^46^ ^]^ and thus, this may not reflect the human situation well, in which T2DM is often accompanied by obesity. In addition to hyperglycemia, diabetic TH mice showed higher serum levels of triglycerides and insulin compared to both control groups. We did not evaluate the presence or size of beta cells in the pancreas. Although the insulin level may be as a surrogate for beta‐cell function it would be interesting to explore this aspect in the future. Because previous studies have only conducted histology of pancreatic islet of diabetic TH mice and shown hypertrophy, hyperplasia followed by cell degranulation compared to SWR/J.^[^ ^29^ ^]^ In addition, they displayed elevated levels of cholesterol and urea, although this is not likely related to the development of diabetes but rather the strain, as nondiabetic TH mice had similar levels as diabetic TH mice.

Impaired glucose tolerance is well‐recognized as a precursor to the development of T2DM in humans. The progression from glucose intolerance to diabetes is known to be accompanied by failure of insulin secretion in response to glucose.^[^ ^2^ ^]^ The intraperitoneal blood glucose and insulin tolerance test revealed impaired glucose tolerance and insulin resistance in high‐TH compared to both SWR/J and low‐TH. Hence, it seems that insulin resistance is an initial malfunction observed in high‐TH mice. As the diabetic stage progresses, inadequate β‐cell compensation results in glucose intolerance and T2DM in high‐TH mice. Our findings align with previous studies showing that TH mice with hyperglycemia showed the onset of persistent hyperglycemia occurring at 10 weeks of age. Moreover, body weight was significantly higher when compared to SWR/J.^[^ ^33^ ^]^ Another study used C57BL/6J as a nondiabetic control. Male TH mice with diabetes showed signs of obesity with a higher body weight at 4 weeks of age. Hyperglycemia was present at 10 weeks of age with 13.8 mmol/L compared to C57BL/6J.^[^ ^29^ ^]^ Because SWR/J and C57BL/6J are not proper wild‐type controls another study investigated the metabolic phenotype in male TH mice with and without T2DM. Male TH mice with T2DM had a significantly higher blood glucose along with elevated HbA1c and a lower serum concentration compared to TH mice without T2DM.^[^ ^35^ ^]^ We did not assess the circulating levels of leptin nor the food intake, which would have been an intriguing aspect to elucidate any disparities that could potentially account for the observed impact on glucose tolerance. However, according to the literature one prior study did examine the food intake in diabetic TH male mice in comparison to C57BL/6 mice. They reported that the food intake rate was 15% higher in diabetic TH male mice compared to C57BL/6J. Furthermore, another study evaluated fasting and nonfasting plasma leptin levels. They showed increased levels of both fasting and nonfasting levels of leptin in diabetic TH male mice compared to C57BL/6 mice.^[^ ^47^, ^48^ ^]^ Therefore, this would be considered as one limitation in our study.

In summary, our findings show TH mice exhibit mild obesity, hyperglycemia, and impaired glucose tolerance, along with insulin resistance compared to both SWR/J and the nondiabetic strain‐matched control replicating metabolic abnormalities observed in human T2DM.

We next characterized the skeletal phenotype of TH mice. With regard to the femur, cortical BV/TV and thickness was higher in high‐TH male mice only in comparison to the SWR/J mice and is consistent with previous studies.^[^ ^33^ ^]^ This was further established for mice at both 8 and 17 weeks of age. In TH mice, the cortical tissue exhibits a higher mineral:matrix ratio (ν1PO4/Proline and ν1PO4/Amide I), hence greater cortical parameters compared to SWR/J.^[^ ^33^, ^34^, ^36^ ^]^ Other rodent models of T2DM including the KK/AY mice show similar trends of increased cortical tissue mineralization.^[^ ^49^, ^50^ ^]^

In contrast another study compared structural properties in the femur of the diaphysis in TH either with or without T2DM and showed significant reduction in cortical area and thickness stating it as an evidence of diabetic bone phenotype in TH model.^[^ ^35^ ^]^ However, cortical thickness is usually increased in patients with T2DM at peripheral sites measured by high‐resolution peripheral quantitative computed tomography (HR‐pQCT).^[^ ^51^ ^]^ In our study, we did not find any differences in terms of cortical parameters between both subgroups of TH male mice. A limitation of our study is the lack of an assessment of calcium deposition between the two subgroups of TH mice. It would have been interesting to explore whether any disparities in calcium deposition between these groups could shed additional light on cortical porosity. TH mice exhibited severe trabecular bone deficit compared to SWR/J. Trabecular parameters in male TH mice with T2DM were also lower when compared to nondiabetic C57BL/6J mice.^[^ ^29^, ^32^, ^52^ ^]^ However, in our study we did not observe notable differences comparing high‐TH to low‐TH mice. Of note, another study using a very large number of mice per group (diabetic *n* = 43 and nondiabetic *n* = 16 has reported lower bone trabecular parameters comparing diabetic to nondiabetic TH mice.^[^ ^35^ ^]^ The observed lower trabecular deficits could be attributed to excessive bone resorption caused by increased osteoclast activities in TH mice, without adequate compensation from bone formation.^[^ ^33^ ^]^ Moreover, the femur length revealed to be shorter for SWR/J compared to both subgroups of TH mice and indicate a correlation between bone traits and body mass.^[^ ^53^ ^]^

Earlier investigations have described femoral three‐point bending showing substantial evidence between TH mice with a higher maximum load, but less post‐yield deformation compared to its nondiabetic TH control.^[^ ^35^ ^]^ When comparing TH mice with SWR/J, studies have reported structural differences in terms of strength which was higher for diabetic TH mice whereas post‐yield displacement was lower,^[^ ^33^ ^]^ further indicating the mid‐femoral diaphysis is stronger but more brittle hence the increased risk of bone fragility. Moreover, another study revealed lower post‐yield displacement for TH mice but maximum moment, stiffness, and work to fracture were similar for both TH mice and C57BI/6J.^[^ ^54^ ^]^ Our three‐point bending of femurs indicated no change for maximum load or energy to failure in any of the three groups. On the other hand, we saw a difference with our compression test revealing high‐TH mice showed lower energy to failure compared to SWR/J. Further comparing TH mice with and without hyperglycemia showed reductions in stiffness, yield force, and ultimate force although overall resistance to fracture showed no change between the two subgroups.^[^ ^35^ ^]^

The difference observed for the vertebrae suggests that the trabecular bone in vertebrae exhibits different mechanical properties compared to femoral bone.

It is worth noting that the trabecular bone in different parts of the body may have varying microarchitecture and mechanical behavior due to functional differences, so the results of the femoral three‐point bending may not directly apply to the spinal trabecular bone. Moreover, the reduced ductility at the whole bone level could potentially be explained by alterations in mineral or the matrix properties in the bone.^[^ ^54^ ^]^

However, further research is needed to better understand the mechanical properties and behavior of spinal trabecular bone.

Finally, we observed no major differences between histomorphometric parameters of bone remodeling or serum parameters of bone turnover in diabetic versus nondiabetic TH mice. Only bone turnover markers were lower in TH mice compared to SWR/J mice, indicating that the SWR/J strain per se might have a higher state of bone turnover. Along those lines, bone marrow adipocyte numbers were higher in both TH groups compared to SWR/J mice, but not different between the TH groups, again suggesting that strain differences may account for these effects.

With the silver nitrate staining we showed that number of osteocyte lacunar and dendrites were higher specifically in the trabecular bone in the femur for the SWR/J compared to high‐TH and low‐TH, although no change observed comparing both subgroups of TH mice. Further investigations have also indicated that the osteocyte lacunar network is adversely affected by T2DM and that the density of lacunar volume is decreased.^[^ ^55^, ^56^, ^57^ ^]^ With the comprehensive 3D evaluation using the ultra‐high‐resolution‐μCT revealed a higher lacunar volume and density for SWR/J compared to both TH subgroups in the trabecular bone in the femur and vertebral body. A similar trend was observed pertaining the lacunar volume and lacunar density in the cortical compartment. These findings are in line with our observations when comparing high‐TH with SWR/J. On the contrary, another study revealed that lacunar density was increased in STZ‐induced hyperglycemia rats.^[^ ^23^ ^]^ Along with yet another study that reported no change in the overall number of dendrite processes in animals with T2DM.^[^ ^26^ ^]^

Further, the osteocyte morphology revealed the osteocyte lacunar being much less spherical and showed significant signs of being more stretched compared to SWR/J. The observed trend was evident across trabecular and cortical compartments both in the femur and in the spine. These strong differences were apparent only comparing high‐TH to SWR/J. Previous studies have indicated a correlation between low BV/TV and spherical lacunar. Possibly osteocytes can remodel in weak bone regions, forming spherical lacunar morphology as a compensatory mechanism to enhance mechanical signaling. This suggests that increased sphericity indicates reduced sensitivity and lower bone quality.^[^ ^58^ ^]^ More importantly, the fact that we found no differences in our lacunar morphometric analysis between TH with hyperglycemia and without hyperglycemia further strengthens the argument that the TH polygenic mouse model is not a proper model for diabetic bone disease because the genetic background may be responsible for the changes we observed.

In summary, high‐TH mice displayed bone alterations only when compared to SWR/J, possibly due to the different size and geometry of the mouse strains. Our data suggest that previously published differences might therefore stem from the genetic background rather than the hyperglycemia state. In conclusion, our study sheds light on the metabolic and skeletal characteristics of TH with and without T2DM and the recommended control SWR/J. Although TH mice may be suitable to study mechanisms of T2DM development, they may not be an ideal model to study diabetic bone disease, as they showed no bone changes compared with their strain‐matched nondiabetic controls.

## Author Contributions

**Lejla Emini:** Data curation; formal analysis; investigation; methodology; resources; validation; visualization; writing – original draft. **Juliane Salbach‐Hirsch:** Conceptualization; supervision; writing – review and editing. **Johannes Krug:** Formal analysis; investigation; software. **Katharina Jähn‐Rickert:** Software; writing – review and editing. **Björn Busse:** Software; writing – review and editing. **Martina Rauner:** Conceptualization; funding acquisition; project administration; supervision; visualization; writing – original draft; writing – review and editing. **Lorenz Hofbauer:** Conceptualization; funding acquisition; project administration; supervision; visualization; writing – review and editing.

## Disclosures

Lorenz C. Hofbauer reports honoraria from advisory boards from Amgen and UCB to himself and support from clinical trials from Ascendis to his institution. Martina Rauner reports honoraria from UCB. Juliane Salbach‐Hirsch is supported by the German Research council: DFG SFB‐TRR67 59307082 subproject B2. She was further supported by the MeDDrive and the women's Habilitation program of the medical faculty of the TU Dresden. All other authors have nothing to declare.

## Supporting information


**Fig. S1.** Elevated triglyceride, urea, cholesterol and insulin in hyperglycemic male TH mice. Serum was collected from 12‐week‐old male TallyHo/JngJ with or without T2DM and the recommended control SWR/J. (A) Triglyceride, (B) Cholesterol, (C) Urea and (D) Insulin, were measured by ELISA. Data are shown as mean ± SD. *n* = 12. Statistical analysis was performed by one‐way ANOVA followed by Bonferroni correction. Statistical significance is denoted in the graphs. *p < 0.05, **p < 0.001, ***p < 0.001.

**Fig. S2.** Cortical and trabecular parameters. Bones from 12‐week‐old male TallyHo/JngJ either with hyperglycemia and without hyperglycemia and SWR/J were examined by microCT. (A) cortical bone mineral density (Ct.BMD) at the femoral midshaft was determined. (B) trabecular thickness (Tb.Th) in the femur and (C) trabecular thickness (Tb.Th) the L4 vertebrae were evaluated from all three groups. Data are shown as mean ± SD. *n* = 16. Statistical analysis was performed by one‐way ANOVA and Bonferroni. Statistical significance is denoted in the graphs. *p < 0.05, **p < 0.001, ***p < 0.001.

**Fig. S3.** Differences in trabecular bone architecture and bone mineral density. Bones from 20‐week‐old male TallyHo/JngJ either with hyperglycemia and without hyperglycemia and SWR/J were examined by microCT. (A) cortical bone volume/total volume (Ct.BV/TV) and (B) cortical thickness at the femoral midshaft was determined (C) bone volume per total volume (BV/TV), (D) trabecular separation (Tb.Sp) and (E) trabecular number (Tb.N) were determined in the distal femur. (F) bone volume per total volume (BV/TV), (G) trabecular spacing (Tb.Sp) and (H) trabecular number (Tb.N) were evaluated at L4 vertebrae from all groups. Data are shown as mean ± SD. *n* = 16. Statistical analysis was performed by one‐way ANOVA and Bonferroni. Statistical significance is denoted in the graphs. *p < 0.05, **p < 0.001, ***p < 0.001.

**Fig. S4.** Bone turnover markers. Serum samples from 20‐week‐old TallyHo/JngJ that either are diabetic and nondiabetic and the recommended control SWR/J were used to determine bone turnover markers. (A) bone resorption marker C‐terminal telopeptide (CTX) (B) bone formation marker type 1 procollagen amino‐terminal‐propeptide (P1NP) and (C) Tartrate Resistant Acid Phosphatase (TRAcP5b) were measured by ELISA. Data are shown as mean ± SD. (CTX *n* = 5–10; P1NP *n* = 5–8; TRAcP5b *n* = 5–9). Statistical analysis was performed by one‐way ANOVA and followed by Bonferroni. Statistical significance is denoted in the graphs. *p < 0.05, **p < 0.001, ***p < 0.001.

**Fig. S5.** The osteocyte canaliculi network is only impaired in the vertebrae. (A) number of osteocytes and (B) number of dendrites in the cortex of the L6 vertebrae including (C) number of osteocytes and (D) number of dendrites in the trabecular bone of the L6 vertebrae were assessed by silver nitrate staining. Data are shown as mean ± SD. *n* = 7. Statistical analysis was performed by one‐way ANOVA and Bonferroni. Statistical significance is denoted in the graphs. *p < 0.05, **p < 0.001, ***p < 0.001.

**Fig. S6.** Characterization of osteocyte lacunae network. High‐resolution microCT was performed on vertebrae from 12‐week‐old TALLYHO/JngJ either with or without T2DM and the recommended control SWR/J. (A) Trabecular lacunae volume over number of lacunae (Tb. Lc.V/N.Lc) and (B) Trabecular lacunae volume over bone volume (Tb. Lc.V/Tb.BV). (C) Trabecular lacunae sphericity (Tb. Lc.Sph) and (D) trabecular lacunae stretch (Tb. Lc.St). Data are shown as mean ± SD. *n* = 7. Statistical analysis was performed by one‐way ANOVA and Tukey. Statistical significance is denoted in the graphs. *p < 0.0332, **p < 0.0021, ***p < 0.0002, ****p < 0.0001.

## Data Availability

The authors confirm that the data supporting the findings of this study are available within the article and its Supplementary material.
