# Supplementary figures and images for: Utility and Limitations of TALLYHO/JngJ as a Model for Type 2 Diabetes–Induced Bone Disease
Source: JBMR Plus. 2023 Nov 17;7(12):e10843. doi: 10.1002/jbm4.10843 (PMC10731141; doi:10.1002/jbm4.10843)

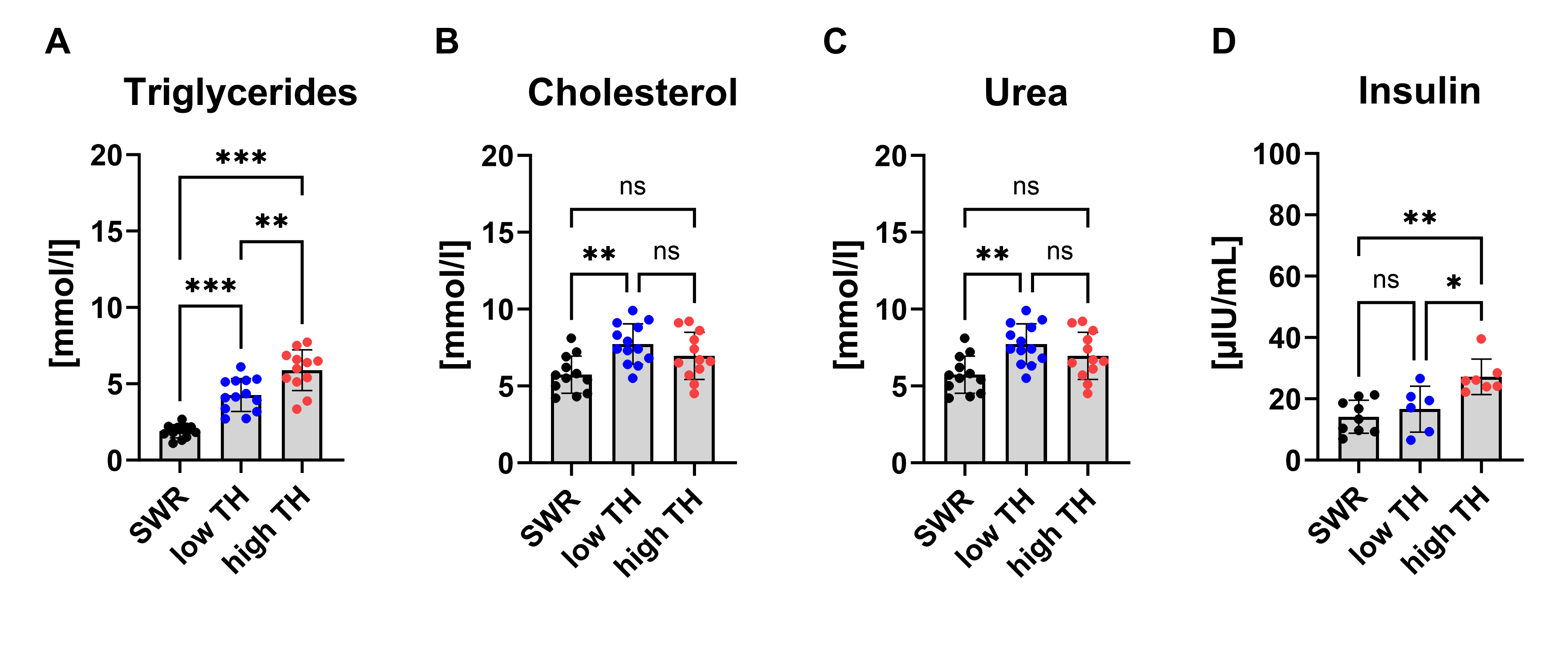

Supplement: Supplementary file 1 — Fig. S1. Elevated triglyceride, urea, cholesterol and insulin in hyperglycemic male TH mice. Serum was collected from 12‐week‐old male TallyHo/JngJ with or without T2DM and the recommended control SWR/J. (A) Triglyceride, (B) Cholesterol, (C) Urea and (D) Insulin, were measured by ELISA. Data are shown as mean ± SD. n = 12. Statistical analysis was performed by one‐way ANOVA followed by Bonferroni correction. Statistical significance is denoted in the graphs. *p < 0.05, **p < 0.001, ***p < 0.001. [file JBM4-7-e10843-s005.tif]

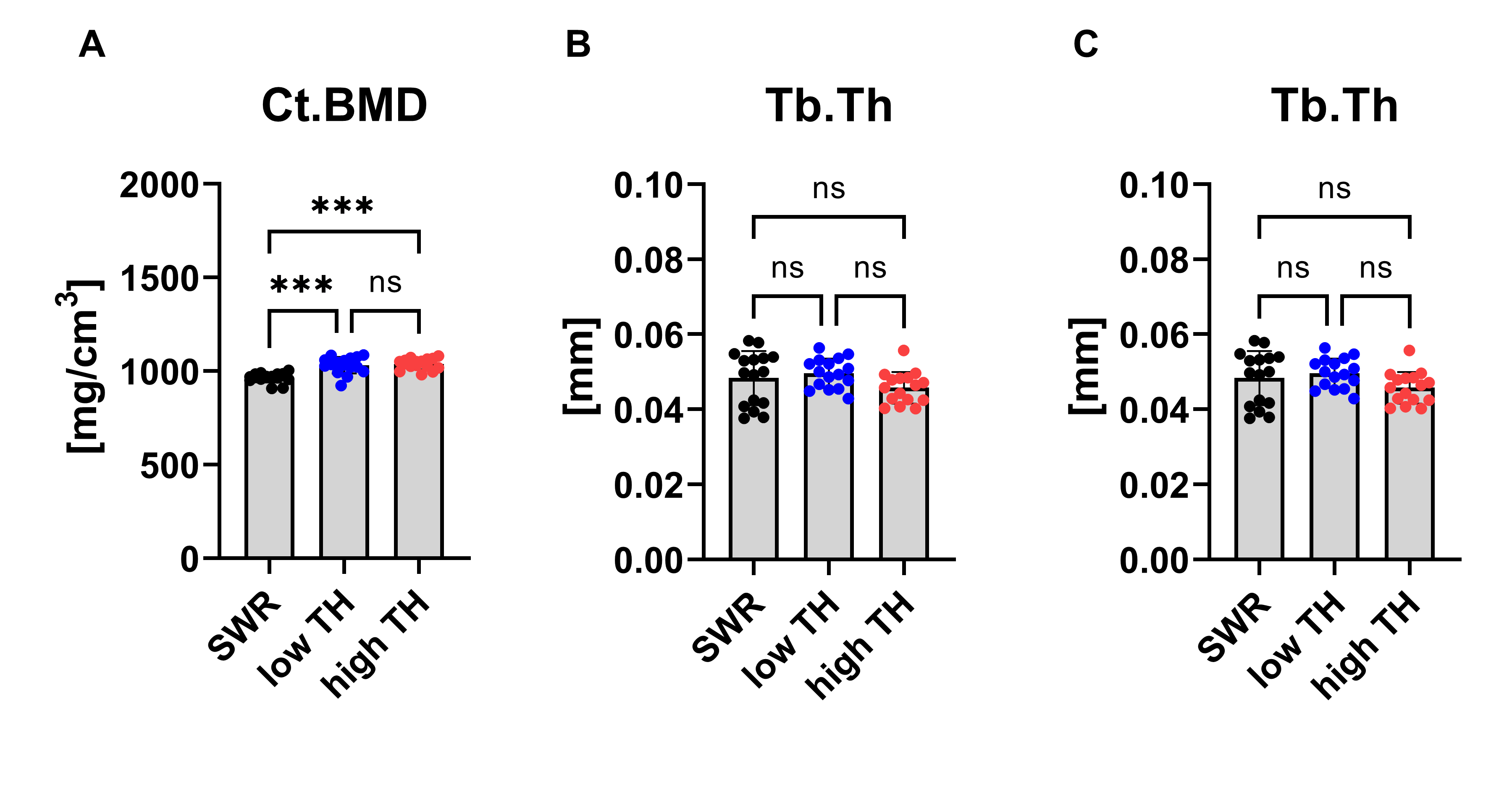

Supplement: Supplementary file 2 — Fig. S2. Cortical and trabecular parameters. Bones from 12‐week‐old male TallyHo/JngJ either with hyperglycemia and without hyperglycemia and SWR/J were examined by microCT. (A) cortical bone mineral density (Ct.BMD) at the femoral midshaft was determined. (B) trabecular thickness (Tb.Th) in the femur and (C) trabecular thickness (Tb.Th) the L4 vertebrae were evaluated from all three groups. Data are shown as mean ± SD. n = 16. Statistical analysis was performed by one‐way ANOVA and Bonferroni. Statistical significance is denoted in the graphs. *p < 0.05, **p < 0.001, ***p < 0.001. [file JBM4-7-e10843-s003.tif]

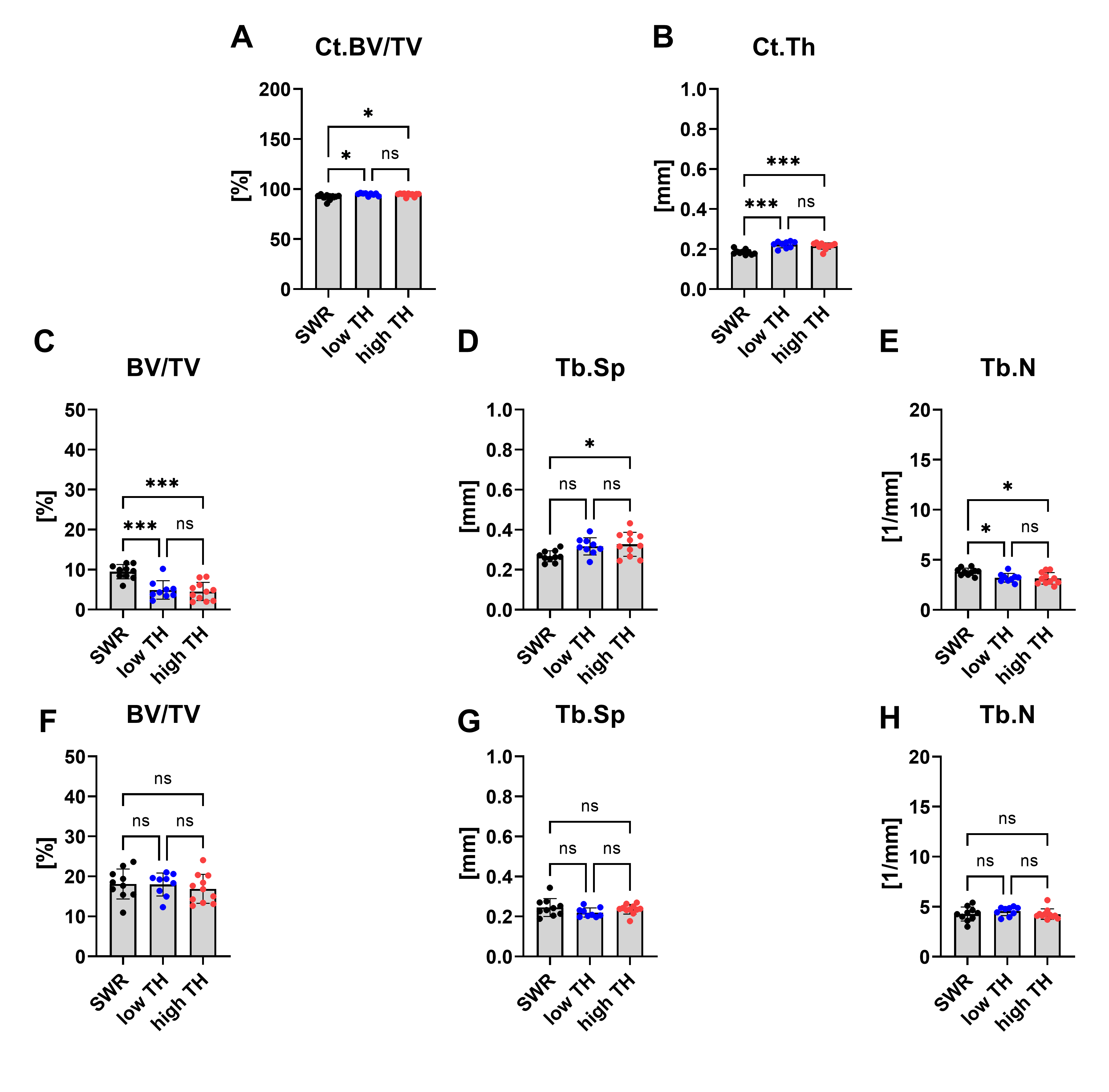

Supplement: Supplementary file 3 — Fig. S3. Differences in trabecular bone architecture and bone mineral density. Bones from 20‐week‐old male TallyHo/JngJ either with hyperglycemia and without hyperglycemia and SWR/J were examined by microCT. (A) cortical bone volume/total volume (Ct.BV/TV) and (B) cortical thickness at the femoral midshaft was determined (C) bone volume per total volume (BV/TV), (D) trabecular separation (Tb.Sp) and (E) trabecular number (Tb.N) were determined in the distal femur. (F) bone volume per total volume (BV/TV), (G) trabecular spacing (Tb.Sp) and (H) trabecular number (Tb.N) were evaluated at L4 vertebrae from all groups. Data are shown as mean ± SD. n = 16. Statistical analysis was performed by one‐way ANOVA and Bonferroni. Statistical significance is denoted in the graphs. *p < 0.05, **p < 0.001, ***p < 0.001. [file JBM4-7-e10843-s002.tif]

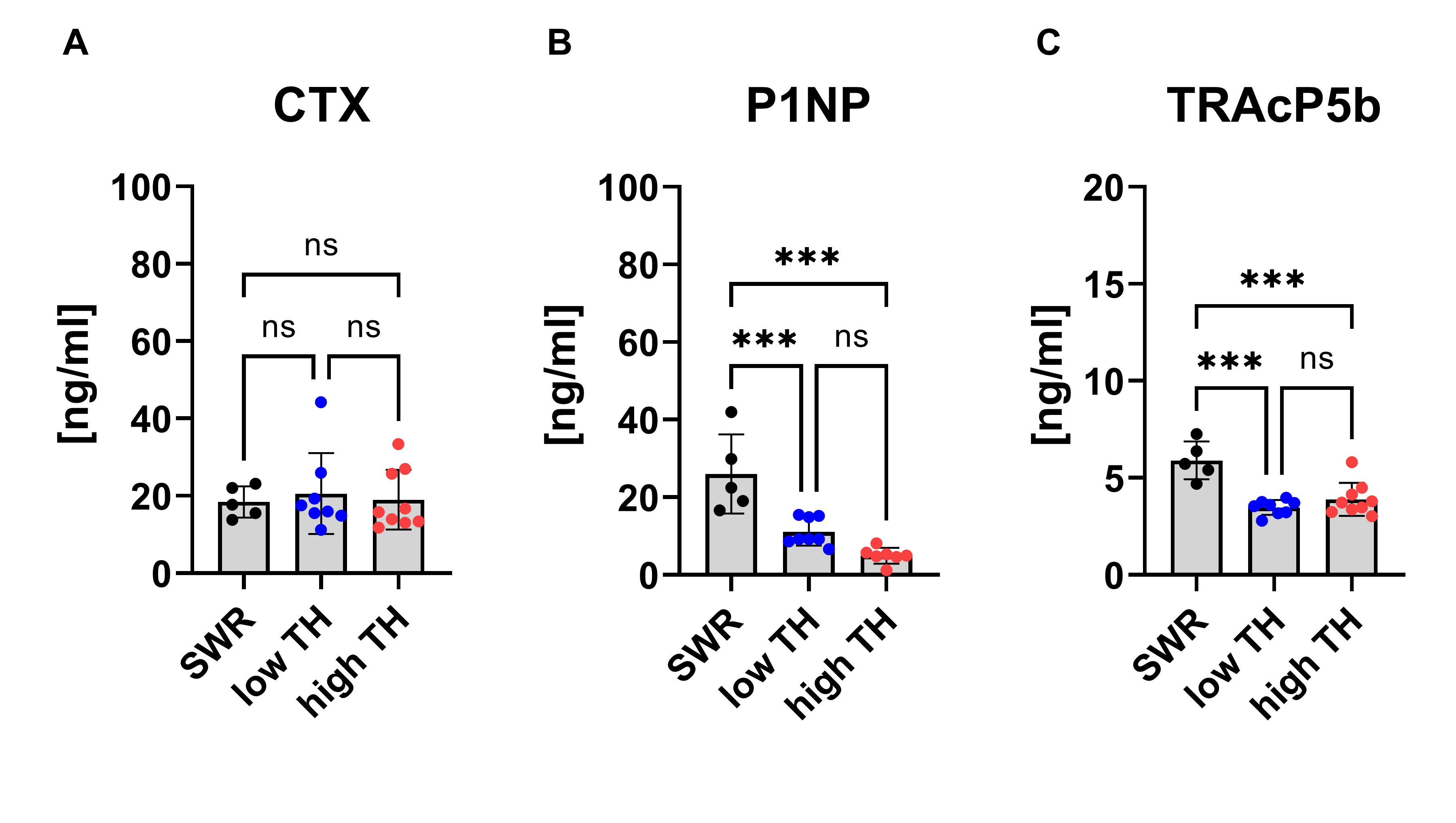

Supplement: Supplementary file 4 — Fig. S4. Bone turnover markers. Serum samples from 20‐week‐old TallyHo/JngJ that either are diabetic and nondiabetic and the recommended control SWR/J were used to determine bone turnover markers. (A) bone resorption marker C‐terminal telopeptide (CTX) (B) bone formation marker type 1 procollagen amino‐terminal‐propeptide (P1NP) and (C) Tartrate Resistant Acid Phosphatase (TRAcP5b) were measured by ELISA. Data are shown as mean ± SD. (CTX n = 5–10; P1NP n = 5–8; TRAcP5b n = 5–9). Statistical analysis was performed by one‐way ANOVA and followed by Bonferroni. Statistical significance is denoted in the graphs. *p < 0.05, **p < 0.001, ***p < 0.001. [file JBM4-7-e10843-s004.tif]

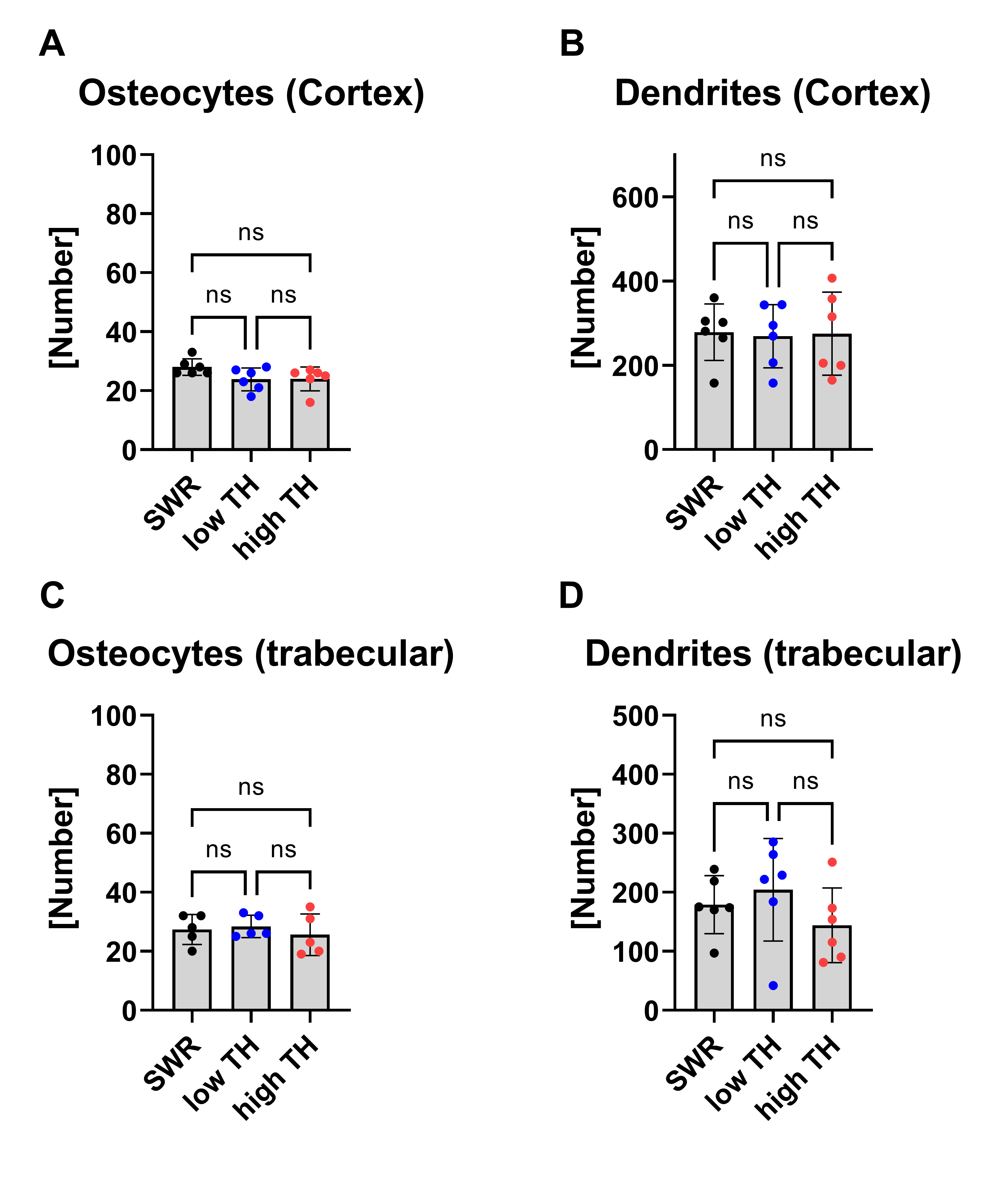

Supplement: Supplementary file 5 — Fig. S5. The osteocyte canaliculi network is only impaired in the vertebrae. (A) number of osteocytes and (B) number of dendrites in the cortex of the L6 vertebrae including (C) number of osteocytes and (D) number of dendrites in the trabecular bone of the L6 vertebrae were assessed by silver nitrate staining. Data are shown as mean ± SD. n = 7. Statistical analysis was performed by one‐way ANOVA and Bonferroni. Statistical significance is denoted in the graphs. *p < 0.05, **p < 0.001, ***p < 0.001. [file JBM4-7-e10843-s006.tif]

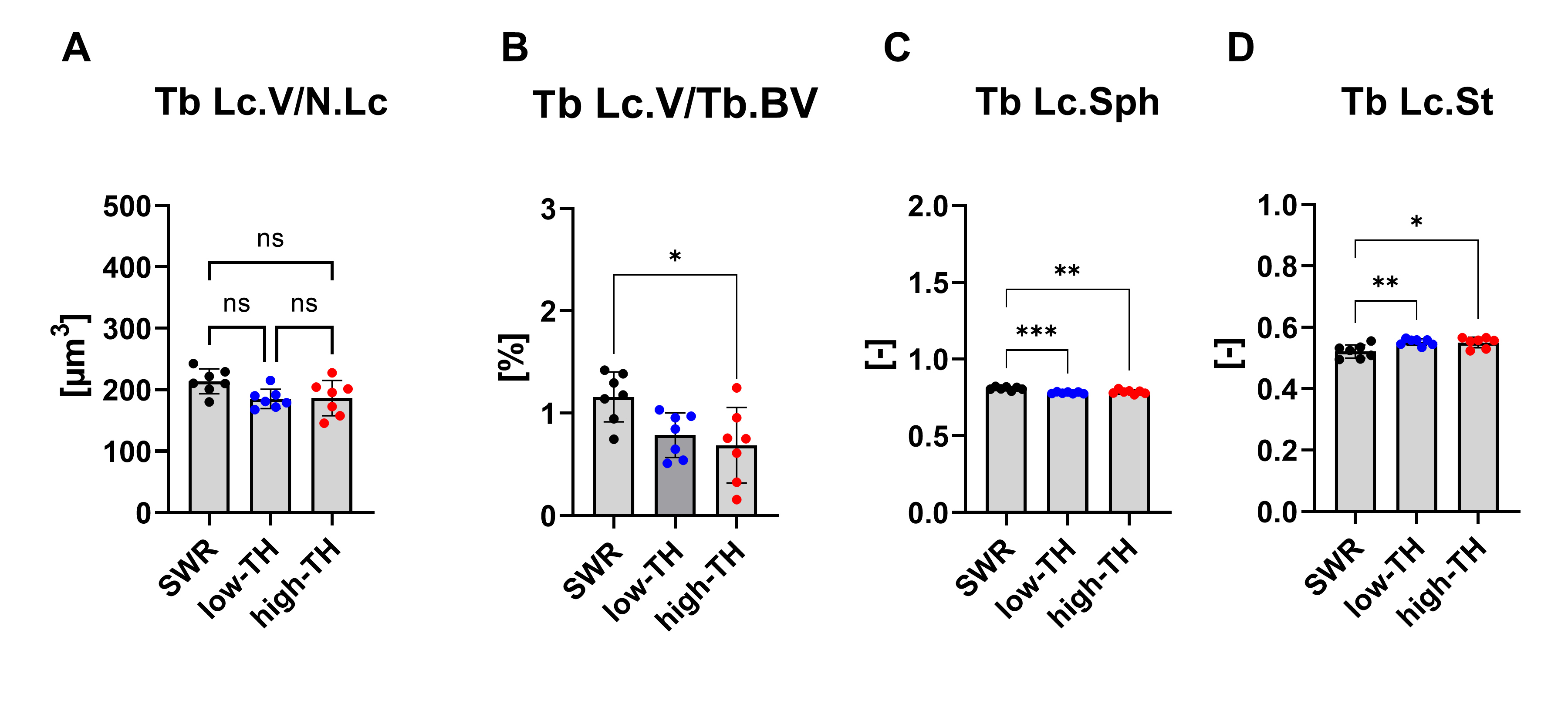

Supplement: Supplementary file 6 — Fig. S6. Characterization of osteocyte lacunae network. High‐resolution microCT was performed on vertebrae from 12‐week‐old TALLYHO/JngJ either with or without T2DM and the recommended control SWR/J. (A) Trabecular lacunae volume over number of lacunae (Tb. Lc.V/N.Lc) and (B) Trabecular lacunae volume over bone volume (Tb. Lc.V/Tb.BV). (C) Trabecular lacunae sphericity (Tb. Lc.Sph) and (D) trabecular lacunae stretch (Tb. Lc.St). Data are shown as mean ± SD. n = 7. Statistical analysis was performed by one‐way ANOVA and Tukey. Statistical significance is denoted in the graphs. *p < 0.0332, **p < 0.0021, ***p < 0.0002, ****p < 0.0001. [file JBM4-7-e10843-s001.tif]
